# RETRACTION: Effect of intra‐wound vancomycin on the surgical site wound infection after spinal surgery: A meta‐analysis

**DOI:** 10.1111/iwj.70129

**Published:** 2024-11-12

## Abstract

**RETRACTION**: YangC. , WangQ. , ZhaoW. , “Effect of intra‐wound vancomycin on the surgical site wound infection after spinal surgery: A meta‐analysis,” International Wound Journal 21, no. 2 (2023): e14653, 10.1111/iwj.14653.PMC1084739242052912

The above article, published online on 06 February 2024, in Wiley Online Library (http://onlinelibrary.wiley.com/), has been retracted by agreement between the journal Editor in Chief, Professor Keith Harding; and John Wiley & Sons, Ltd. The authors listed for this article were changed post‐acceptance. The publisher was contacted by individuals claiming to be the original authors and was notified that their email account had been compromised, which lead to the change at the listed authorship. The identity of the individuals who contacted the journal could not be confirmed. Following additional investigation of the peer review process for this article, the parties have concluded that this article was originally accepted solely on the basis of a compromised peer review process. Therefore, the article must be retracted. The listed authors disagree with the retraction.

**RETRACTION**: C. Yang , Q. Wang , W. Zhao , “Effect of intra‐wound vancomycin on the surgical site wound infection after spinal surgery: A meta‐analysis,” International Wound Journal 21, no. 2 (2023): e14653, 10.1111/iwj.14653.PMC1084739242052912

The above article, published online on 06 February 2024, in Wiley Online Library (http://onlinelibrary.wiley.com/), has been retracted by agreement between the journal Editor in Chief, Professor Keith Harding; and John Wiley & Sons, Ltd. The authors listed for this article were changed post‐acceptance. The publisher was contacted by individuals claiming to be the original authors and was notified that their email account had been compromised, which lead to the change at the listed authorship. The identity of the individuals who contacted the journal could not be confirmed. Following additional investigation of the peer review process for this article, the parties have concluded that this article was originally accepted solely on the basis of a compromised peer review process. Therefore, the article must be retracted. The listed authors disagree with the retraction.

